# Anaphylactic reaction in patient allergic to mango

**DOI:** 10.1186/s13223-018-0294-1

**Published:** 2018-10-31

**Authors:** Natalia Ukleja-Sokołowska, Ewa Gawrońska-Ukleja, Kinga Lis, Magdalena Żbikowska-Gotz, Łukasz Sokołowski, Zbigniew Bartuzi

**Affiliations:** 1 Department and Clinic of Allergology, Clinical Immunology and Internal Diseases, Ludwik Rydygier Collegium Medicum in Bydgoszcz, NCU, Bydgoszcz, Poland; 2 Division of Ergonomics and Exercise Physiology, Department of Hygiene, Epidemiology and Ergonomics, Ludwik Rydygier Collegium Medicum in Bydgoszcz, NCU, Bydgoszcz, Poland

**Keywords:** Mango, Allergy, Mugwort, Inhibition test, Art v 1

## Abstract

**Background:**

An allergy to mango is extremely rare. The antigenic composition of the fruit is not fully known. Profilin from mango has a structure similar to birch tree profiling: it is responsible for cross-reactions between mango and pear, apple, and peach. A panallergen with a structure similar to mugwort defensin (Art v 1) which cross-reacts with celery, carrot, peanuts, pepper, aniseed, and caraway has been previously described.

**Case study:**

A female patient, 30 years old, was admitted in February 2017 because of recurrent allergic reactions following consumption of various foods. The most severe allergic reaction in the patient’s life occurred after eating a mango fruit. Within several minutes the patient developed a generalised urticaria, followed by facial oedema, strong stomach pain and watery diarrhoea. The diagnostics involved skin tests with a set of inhalatory and food allergens, including native skin tests. The patient also experienced symptoms of recurrent, generalized urticaria in connection with consumption of various types of food, especially complex dishes containing many different ingredients. Additionally, an interview revealed that the patient was experiencing symptoms of the oral allergy syndrome after ingesting various fruit and vegetables, especially during late summer and fall. Diagnostics was extended by determining the levels of IgE specific for allergen components, using the ImmunoCap ISAC method. In order to confirm the occurence of a cross-reaction between mugwort and mango allergens, we performed the inhibition test of IgE specific for mugwort using a mango allergen extract and ImmunoCap matrix.

**Results:**

Skin prick tests (SPT) were positive for allergens of grass 7 mm; weeds 8 mm; cat’s fur 5 mm; mugwort 6 mm. SPT were also positive for mango. The level of specific IgE was increased for allergens of mugwort, grass, celery, pepper, carrot, mango, banana, peach, and apple. The ImmunoCap ISAC test demonstrated a high level of specific IgE rPhl p 1 (timothy grass) and Art v 1 (mugwort). We also performed the IgE inhibition test using both mango extract and ImmunoCap matrix and confirmed a cross-reaction with Art v 1 in the pathogenesis of symptoms observed in the patient.

**Conclusions:**

An anaphylactic reaction to consumed mango, resulting from cross-allergy with mugwort Art v 1 was diagnosed in the patient. Acute urticarial in this case is a manifestation of IgE-mediated food allergy. During in vitro diagnostic procedures we found an elevated concentration of IgE specific to several food allergens (including celery, peppers, carrot, banana, peach, apple, shrimp). The elimination diet removing allergens the patient was allergic to was recommended. Considering the anaphylactic reaction the patient was instructed to carry a rescue set composed of an adrenaline autosyringe, steroids, and antihistamines.

## Background

Mango (Latin: *Mangifera indica*), called the “king of fruit”, is one of the most commonly grown exotic fruits. It originally comes from South Asia. The plant belongs to the *Anacardiaceae* family. Other representatives of the same family are pistachios and cashew nuts [1].

Mango has been cultivated for approximately 6000 years. In India the fruit is referred to as the “fruit of the gods”. It is the most important fruit in India. Mango constitutes half of the whole fruit production in India.

Indian mango is a popular edible fruit. The fruit is a drupe, with a large pit. Mango contains a glycoside called mangiferin which possesses anti-viral, anti-microbial, anti-atherosclerotic, and anti-diabetic properties. The fruit is also a rich source of beta carotene and vitamin C [2].

An allergy to mango is extremely rare. Hypersensitivity to mango allergens may be immediate or delayed. In 2011 Sareen et al. performed a meta-analysis of cases of allergic reactions to mango available in the Medline/Pubmed database. They found 17 papers concerned with a total of 22 patients. Ten of these patients had immediate reactions, and the rest had delayed reactions. The most common symptoms included: wheezing, urticaria, angioedema, and anaphylaxis [3, 4].

The antigenic composition of the fruit is not fully known. 334 different proteins were found in mango peel, and 2855 in the fruit flesh. Some of these proteins may possess immunizing properties [5].

The following mango allergens have been identified to date:

- Man i 1—the major allergen, molecular weight of 40 kDa and unknown function;
- Man i 2—the major allergen, molecular weight of 30 kDa and unknown function;
  - Little is known about the biological role of those allergens. In 2017 Tsai et al. [6] described the sequence of Man i 1 and its expression protocol and subsequent replication in *Escherichia coli* cell during mitosis.
- Man i 3—a minor allergen, a cross-reacting profilin [7]—a panallergen with a structure similar to mugwort defensin (Art v 1) which cross-reacts with celery, carrot, peanuts, pepper, aniseed, and caraway was also described in this study. It was discovered that allergens from mango may cross-react with latex allergens, through the phenomenon known as the latex-fruit syndrome [4, 7].

Mugwort (Latin *Artemisia vulgaris*) is the most important representative of the *Asteraceae family.* It is one of the main causes of allergic reactions in Europe. It is estimated that approximately 95% of patients allergic to mugwort have IgE against Art v 1, the main allergen, a glycoprotein with a defensin-like domain [8, 9].

## The case study

A female patient, 30 years old, was admitted in February 2017 to the Department and Clinic of Allergology, Clinical Immunology and Internal Diseases because of recurrent allergic reactions following consumption of various foods.

These reactions were largely dermal, in the form of urticaria on the whole body. Symptoms of recurrent, generalized urticaria were experienced after ingesting various especially complex dishes containing many different ingredients. The patient associated those symptoms with consumption of peppers, clementines, buckwheat honey, and red wine.

The most severe allergic reaction in the patient’s life occurred after eating a mango fruit. Within several minutes the patient developed a generalised urticaria, followed by facial oedema, strong stomach pain, and watery diarrhoea. Paramedics were called. The patient was administered adrenaline, steroids, and antihistamines.

A detailed medical history revealed that for the previous 6 years the patient had symptoms of seasonal rhinitis (lacrimation, itching eyelids, and watery nasal discharge).

The patient at the time was also being treated with levothyroxine for hypothyroidism. The patient’s family history is ridden with allergic diseases: the patient’s mother is allergic to inhalatory allergens, and her father to birch and cat.

In this complex case diagnosis required a wide range of diagnostic methods. As a part of the diagnostics performed in the Department and Clinic of Allergology, Clinical Immunology and Internal Diseases, the patient had skin prick tests with a set of inhalatory allergens (*Dermatophagoides farinae*, *Dermatophagoides pteronyssinus*, grass/cereals, weeds, *Aspergillus fumigatus*, *Cladosporium herbarum*, cat’s fur, poplar, hazel, alder, birch, mugwort, ribwort plantain) and food allergens (chicken egg, cow milk, tomato, carp, banana, rye flour, wheat flour, peanuts, hazelnut, pork, chicken meat, orange) from Allergopharma-Nexter Sp. z o.o. (Ltd.), as well as native skin tests with fresh foods (buckwheat honey, peppers, mango, clementine, carrot, celery, peanuts, banana, mustard, turmeric, caraway, red wine).

Levels of IgE specific for selected allergens (mugwort, wormwood, early grass mix, late grass mix, celery, peppers, carrot, mango, banana, peach, apple, peanuts, hazelnuts, *Dermatophagoides pteronyssinus*, *Dermatophagoides farinae*, early trees mix, late trees mix, birch, *Cladosporium herbarum*, *Aspergillus fumigatus*, *Alternaria tenius*, cow milk protein, seasoning mix, mustard) were determined with the ImmunoCap (*ThermoFisher Scientific*, *Uppsala*, *Sweden*) system, using the Phadia100 equipment according to the manufacturer’s instructions (*ImmunoCAP specifi IgE*, *fluoroenzymeimmunoassay*, *52*-*5291*-*EN/05*).

The diagnostics were expanded by a determination of the level of IgE specific for allergen components, using the ImmunoCap ISAC method.

Each of these tests have different specificity and sensitivity and, no less importantly, availability and cost. Skin prick tests are considered a fast, inexpensive method with immediate results. Unfortunately, their detection rate in food allergy is limited. In a recent article by Griffiths et al. [10] the detection rates for SPTs (53%) and ISAC (59%) were similar, with a higher detection rate for ImmunoCAP testing (66%). In patients with nut allergies, tests for sensitisation to nuts scored similarly, but with a greater sensitivity (71%) for ImmunoCAP tests than SPT (53%) or ISAC (65%). Therefore we extended the diagnosis in this case to in vitro determination of the concentration of allergen specific IgE. The gold standard in diagnosis of food allergy is still the double blind placebo controlled food challenge. In this case the patient did not consent to provocation challenges.

Skin prick tests were positive for the following allergen extracts (wheal average diameter in millimetres): grass 7 mm; weeds: 8 mm; cat’s fur: 5 mm; mugwort: 6 mm; ribwort plantain: 4 mm; celery: 2 mm; clementine: 2 mm (histamine 4 mm, negative control 0 mm, a result was interpreted as positive in case of a wheal average diameter ≥ 3 mm). No skin reaction was observed for other tested allergen extracts. The total IgE level was 406.53 kU/L.

Results of native tests were positive for mango 5 mm, celery 3 mm.

Results for specific IgE levels tested using the ImmunoCap method are presented in Table 1. Elevated levels of IgE (above 0.35 kU/L) were found against mugwort, wormwood, early grass mix, late grass mix, celery, peppers, carrot, mango, banana, peach, apple, peanuts.

**Table 1:** The list of results for specific IgE levels tested using the ImmunoCap method

No.	Allergen	IgE level (kU/L)
1	Mugwort	144.32
2	Wormwood	82.83
3	Early grass mix	37.14
4	Late grass mix	11.77
5	Celery	3.16
6	Peppers	0.98
7	Carrot	0.96
8	Mango	0.94
9	Banana	0.89
10	Peach	0.87
11	Apple	0.59
12	Peanuts	0.39
13	Hazelnuts	< 0.35
14	*Dermatophagoides pteronyssinus*	< 0.35
15	*Dermatophagoides farinae*	< 0.35
16	Early trees mix	< 0.35
17	Late trees mix	< 0.35
18	Birch	< 0.35
19	*Cladosporium herbarum*	< 0.35
20	*Aspergillus fumigatus*	< 0.35
21	*Alternaria tenius*	< 0.35
22	Cow milk protein	< 0.35
23	Seasonings mix	< 0.35
24	Mustard	< 0.35

After the patient was discharged from the hospital we received results of the ImmunoCap ISAC tests (Table 2). A high level of IgE specific for Art v 1 (defensin) from mugwort and Phl p 1 from timothy was particularly noteworthy. It is worth to emphasize that there were no elevated levels of IgE specific to components of food allergens available in ImmunoCap ISAC. It is possible that symptoms of OAS in this patient resulted from cross reactivity with timothy and mugwort allergens. Symptoms of urticaria may be also result from sensitivity to one of the food allergens mentioned above. The only anaphylactic reaction the in patients life occurred after the consumption of mango, and this hypersensitivity was the one that, we decided, required further diagnosis.

**Table 2:** ImmunoCap ISAC test results. No increased levels of allergen-specific IgE were found for other allergen components included in the ImmunoCap test

Allergen source	Allergen component	Allergen type	IgE level (ISU-E)
Bermuda grass	nCyn d 1	Grass group 1	4.6
Timothy grass	rPhl p 1	Grass group 1	38
Timothy grass	rPhl p 4	Berberine bridge enzyme	2.2
Mugwort	nArt v 1	Defensin	34
Cat	rFel d 1	Uteroglobin	3.3
Anisakis	rAni s 3	Tropomyosin	0.5
Cockroach	nBla g 7	Tropomyosin	0.7
*D. pteronyssinus* (house dust mite)	rDer p 10	Tropomyosin	1
Shrimp	nPen m 1	Tropomyosin	0.4

Here it needs to be clearly stated that the ImmunoCap ISAC test did not indicate if in that particular case the patient’s allergy to mango was a result of a cross-allergy with Art v 1, or an allergy to another molecule, independent from mugwort. ImmunoCap ISAC has no mango allergen components available.

To clarify this, then, the ImmunoCap inhibition test was applied, using the allergen extract from fresh mango fruit. The investigation was based on the SPHIA model (*Single Point Highest Inhibition Achievable Assay*) described by Bernardi et al. in 2011 [11].

The methodology of this inhibition assay is still considered experimental, although similar inhibition assays were applied previously by several authors. For example, in our research unit a case of a patient allergic to sunflower seed was described, where ImmunoCap ISAC inhibition test was used to prove cross reactivity. In the inhibition test Art v 1, Art v 3, and Jug r 3 were inhibited by the protein present in the sunflower seed allergen extract [12].

To perform the inhibition test a ripe mango fruit was bought in a local grocery offering eco food. The fruit was washed, peeled and the pit was removed to obtain the edible part. The protein-containing pulp extract was obtained using methods described previously [13, 14]. Diced fruit was frozen in liquid nitrogen and blended to smooth pulp. The pulp was then mixed with 1 M NaCl in the ratio of 2:1 (2:1, v/v) and left at 2–8 °C for 24 h. After that time the mixture was centrifuged at room temperature at 12,000×*g* for 60 min. The extract was obtained containing 2.35 mg/dL of protein. In the inhibition test the obtained extract was mixed with the patient’s blood serum at the volume ratio of 1:1, and incubated at 2–8 °C for 24 h [11, 14]. A blind sample was simultaneously prepared in order to account for the dilution of the serum. For that purpose, the patient’s blood serum was mixed with 1 M NaCl (1:1, v/v), and then incubated at 2–8 °C for 24 h. After that time, the level of IgE specific for mugwort, Art v1, and mango was determined in the prepared material, both inhibited and diluted, and the inhibition ratio was calculated.

In the inhibition test we achieved the following results—the baseline level of mugwort sIgE in the patient’s serum was 144.32 kU/L. In the blind sample, after diluting it with 1 M NaCl 1:1, the concentration of sIgE was 72.16 kU/L. After incubation of the patient’s serum with a mango allergen extract the level of mugwort IgE was 48.81 kU/L. It was thus shown that the mango allergen extract inhibited the binding of mugwort IgE by 32.38%.

**Fig. 1:**
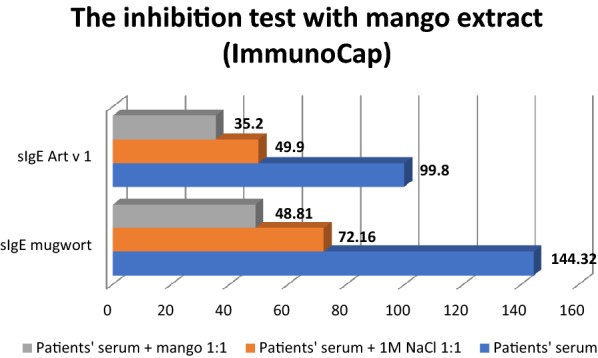
Results of the inhibition test with mango extract

The baseline level of Art v 1 sIgE in the patient’s serum was 99.8 kU/L. In the blind sample, after diluting it with 1 M NaCl 1:1, the concentration of sIgE was 49.9 kU/L. After incubation of the patient’s serum with the mango allergen extract the level of Art v 1 IgE was 35.2 kU/L. It was thus shown that the mango allergen extract inhibited the binding of IgE Art v 1 by 29.5%. Based on the performed diagnostic procedures it was demonstrated that symptoms present in the patient could be a result of a cross-reaction between allergens of mango and Art v 1. Results of the inhibition test are presented in Fig. 1.

Due to the fact that this inhibition test was an experimental method and the previously described protocol was modified to accompany the needs of this specific case, an inhibition test according to a different protocol was performed in order to provide a final confirmation of the cross-allergy. The patient’s serum was incubated with the allergen extract from a mango fruit, using the ImmunoCap matrix. The ImmunoCap was pre-washed four times: twice with the wash worksolution ImmunoCap, and twice with the neutral pH phosphate buffer. 50 µL of tested serum was added to the ImmunoCap prepared that way, and incubated for 1 h at room temperature, and then centrifuged at 1500×*g* for 2 min. The obtained antibody-depleted serum, and the native serum were analysed for the presence of the Art v 1 allergen component, using the ImmunoCap method.

**Fig. 2:**
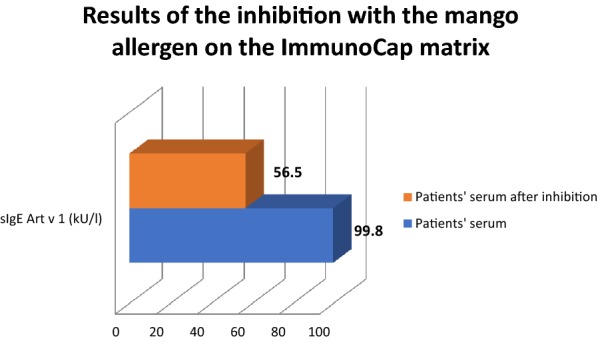
Results of the inhibition with the mango allergen on the ImmunoCap matrix

The baseline level of Art v 1 sIgE in patients serum was 99.8 kU/L. In this case the patient’s serum was not diluted, so negative control was not necessary. After incubation of the patient’s serum with ImmunoCap mango matrix the level of Art v 1 IgE was 56.5 kU/L. As a result it was shown that the ImmunoCap mango matrix inhibited binding of Art v 1 IgE by 43.4%. These results are presented in Fig. 2.

## Discussion

An anaphylactic reaction to consumed mango, resulting from cross-allergy with mugwort Art v 1 was diagnosed in the patient. Allergy to various foods was diagnosed, including celery, peppers, carrot, banana, apple, and peanuts. Allergies to grass pollen, timothy, mugwort, and cat’s fur were also diagnosed.

The presented case was interesting because of a rarely reported anaphylaxis after the consumption of a mango fruit. Mango allergens have not been characterised precisely [6]. According to reports, there are some mango allergens that may cross-react with birch pollen (profilin) and mugwort pollen (defensin). The diagnostics involved inhibition of IgE specific for the mugwort allergen extract and Art v 1, using the mango allergen extract obtained from fresh mango fruit in our laboratory. It was confirmed that the allergen extract inhibited IgE by ~ 30%, which confirmed the participation of the cross-reaction with Art v 1 in the development of symptoms in the analysed patient.

The inhibition test used for confirmation of the cross-allergy as a source of symptoms in a mugwort-allergic patient was described, among others, in 2016: the reported test confirmed a cross-allergy to mugwort Art v 1 and Art v 3 in a patient who developed an anaphylactic reaction following consumption of sunflower seeds [12].

In the present case we used two different protocols for the inhibition assay. Through the first inhibition assay it was shown that the mango allergen extract inhibited the binding of IgE Art v 1 by 29.5%. In the second protocol, the ImmunoCap mango matrix inhibited the binding of Art v 1 IgE by 43.4%. The difference in the inhibition may be with a result of the presence of different antigens in the ImmunoCap Matrix and in the native mango allergen extract, due to different variety of fruit. What is more, ImmunoCap has a matrix with high bioaccessibility and bioavailability, which improves the binding of serum sIgE to antigens.

Art v 1 is a defensin-like protein. It is relatively stable to proteolytic degradation, which may lead to a higher sensitization rate of Art v 1 compering to other defensin-like proteins [e.g. ragweed (*Ambrosia artemisiifolia*) Amb a 4 or Santa Maria feverfew (*Parthenium hysterophorus*) Par h 1] [15]. We might suspect that the mango allergen, cross reactive with Art v 1, is also a stable allergen and for this reason positive results were achieved both during prick by prick test with fresh mango and during immunodiagnostic methods with the mango allergen extract (frozen in liquid nitrogen), and with the ImmunoCap mango allergen extract. We did not perform skin prick tests with a commercial mango allergen extract due to the lack of this allergen in the portfolio of the company from which we acquire extracts for SPT (Allergopharma Nexter).

Reported cases of an allergic reaction to mango are relatevely scarce. In 1942 Kahn described a case of a female patient who “developed some symptoms” after eating a mango fruit. The patient was persuaded to undergo a food challenge test. After eating a mango fruit the patient experienced dyspnoea and wheezing of asthmatic character. Those symptoms disappeared after the administration of adrenaline [16].

In 2009, Silva et al. [17] described a case of a 39-year old female patient who developed an anaphylactic reaction after eating a mango fruit, most probably caused by allergy to the 13 kDa allergen cross-reacting with mugwort allergens, which was confirmed in the *immunoblotting inhibition assay*.

Hedge et al. [1] described an interesting case of a female patient whose symptoms, occurring after consumption of a mango fruit, were most probably a result of an IgE-dependent reaction to a low-molecular-weight protein included in the allergen extract made from a fresh mango fruit. It was observed that similar symptoms occurred in that patient also after ingesting other fruit of the *Anacardiaceae family*, e.g. a cashew fruit (the edible part of the plant with the cashew nut).

In 2017 Valk et al. [8] published an interesting paper analysing 29 cases of children with a confirmed allergy to cashew nuts. The children underwent an oral challenge test with pistachios and mango fruit. None of the children demonstrated hypersensitivity to mango, which indicated a relative low risk of cross-reactions with cashew nuts.

An interesting report was published in 2015 by Ta et al [18]. The authors described a case of an 8-month old patient who experienced recurrent reactions in the form of vomiting, weakness, decreased muscular strength of extremities, and peripheral cyanosis, several hours after consumption of a mango fruit. A detailed diagnostics was performed and an enteritis syndrome caused by a mango protein was diagnosed.

Patients with contact dermatitis involving body parts that had contact with mango pulp were also described [19, 20].

Acute urticaria is a common manifestation of IgE-mediated food allergy, although food allergy is not the most common cause of acute urticaria and is rarely a cause of chronic urticaria [21]. In the case of the patient described here, during in vitro diagnostic procedures we found an elevated concentration of IgE specific to several food allergens (including celery, peppers, carrot, banana, peach, apple, shrimp). The results may be interpreted alongside-cross reactivity to pollen allergens (for example symptoms of oral allergy syndrome) and co-sensitization to food allergens in pollen allergic patients. Moreover, based on the results alone, it is not possible to distinguish between a clinically relevant allergy and asymptomatic sensitisation. An allergen-specific elimination diet, with careful monitoring of symptoms after eliminating 1–2 specific kinds of food was advised in order to improve the patients symptom’s and, at the same time, diagnose the clinically relevant allergy.

According to Boyce et al. [22] elimination of 1 or a few specific foods from the diet may be useful in the diagnosis of food allergy. Although the quality of evidence for this approach is low, it is useful when it is not possible to perform the oral food challenge, the gold standard for diagnosing food allergies. This test is accurate and sensitive, but also presents a greatest risk to the patient. What is more, it is important to remember that prolonged elimination diets that omit multiple foods have been reported to induce severe malnutrition.

Strict elimination of mango, as the cause of anaphylaxis, was advised.

Specific immunotherapy with mugwort allergen extract was recommended to the described patient. Symptomatic treatment of rhinitis was also recommended during pollination seasons. Considering the anaphylactic reaction the patient was also instructed to carry a rescue set composed of an adrenaline autosyringe, steroids, and antihistamines.

On a follow-up visit a detailed interview revealed that the patient did not experience further episodes of anaphylaxis. She strictly avoids mango fruit, and also eliminated celery from her diet. The patient felt that the condition of her skin improved and the episodes of acute urticaria appear not as often and with less intensity. Further observation of symptoms was recommended.

## Summary

The presented case is interesting, because it is one of a few cases of anaphylactic reactions associated with an allergy to a mango fruit. We would like to draw attention to this rare allergen, often discarded during allergy diagnosis, especially due to the lack of commercially available allergen extracts for skin prick testing.

Our particular diagnostic approach is also worth highlighting, because it enabled us to confirm a cross-allergy with mugwort Art v 1 as a potential source of anaphylactic symptoms in the patient. Inhibition testing is a relatively simple tool that allows to distinguish between cross reactivity and co-reactivity.

Patients with inhalatory allergies often have typical symptoms of OAS. There is a group of patients that can experience systemic symptoms as well. In the described case cross reactivity resulted in anaphylaxis. Moreover, the patient had several episodes of generalised urticaria which require further observation. The fact, that patient had elevated IgE to several inhalatory and food allergens makes this case more complicated. In real life conditions many patients suffer simultaneously from allergies of different orgin (for example to LTPs, defensins, PR-10, storage proteins).

The level of knowledge about mango allergens is surely unsatisfactory, and further studies are required. Mango fruit certainly contain cross-reacting allergens, but the presence of species-specific allergens cannot be excluded.

## Abbreviations

SPT: skin prick test
CRD: component resolved diagnosis
